# Advancing cancer therapy with custom-built alternating electric field devices

**DOI:** 10.1186/s42234-024-00164-3

**Published:** 2025-01-30

**Authors:** Isobel Jobson, Nguyen T. N. Vo, Edward Kujawinski, Chris Denning, Snow Stolnik, Veeren M. Chauhan, Frankie Rawson

**Affiliations:** 1 School of Pharmacy, Biodiscovery Institute & Boots Science Building, University of Nottingham, Nottingham, NG7 2RD UK; 2 Division of Cancer & Stem Cells, Biodiscovery Institute, University of Nottingham, Nottingham, NG7 2RD UK; 3 Electronic Workshop, Faculty of Engineering, University of Nottingham, Nottingham, NG7 2RD UK

**Keywords:** Tumour treating fields, Glioblastoma, Electric Fields, Zinc oxide nanoparticles, Cancer therapy

## Abstract

**Background:**

In glioblastoma (GBM) therapy research, tumour treating fields by the company Novocure™, have shown promise for increasing patient overall survival. When used with the chemotherapeutic agent temozolomide, they extend median survival by five months. However, there is a space to design alternative systems that will be amenable for wider use in current research. Therefore, we sought to establish a custom-built alternating electric field device to investigate the effect of electrode design on the responsiveness of cancer cells to this therapy.

**Methods:**

A 96-well microtiter plate modified with an electrode array was fabricated to investigate its application as an in vitro alternating electric field device. This was initially performed with patient-derived GCE 31 and GIN 31 cell lines found in the core and invasive margin of the GBM tumour, respectively. We sought to establish the effect of the application of low-intensity (3 V/ cm) electric fields with an application duration of 4—48 h, using intermediate frequency (300 kHz) alternating currents (AC). To demonstrate that electric fields were entering the cell, GCE 31 and GIN 31 cells were treated with the inorganic, non-conductive zinc oxide (ZnO) nanoparticles (NP), previously demonstrated to enhance the efficacy of TTFs. After a 4-h exposure to NP, cells were then exposed to alternating electric fields or currents and their metabolic activity was assessed. To better understand how the position and morphology of cells can affect cell therapy responsiveness to alternating electric fields or currents, GBM results were compared to those from the semi-adherent brain tumour cell line, D425.

**Results:**

Contrary to previous findings, there was no significant difference between the GIN 31 and GCE 31 cells exposed to alternating electric fields or currents treated with or without NP compared to cells untreated and unstimulated. D425 cells exposed to alternating electric fields exhibited a pronounced metabolic increase (1.8-fold), while those exposed to alternating electric currents with or without ZnO had a reduced metabolism relative to the untreated control.

**Conclusions:**

The initial hypothesis for the lack of effect of electrical stimulation on the adherent cells was that, due to only a single pair of electrodes being used, the proportion of cells that were in the correct orientation for electric field effects was limited. However, the dramatic shift in cell behaviour of the semi-adherent cells shows that cell morphology plays an important role in the responsiveness of cancer cells to AC electric fields. This study highlights the lack of understanding of the complex mechanisms by which electric fields exert effects on cancer cells. We propose that, for the therapy to be enhanced for patients, research should first focus on the underlying mechanisms of action, specifically on how individual cancer cell types respond to this therapy.

## Background

Glioblastoma (GBM) is the most common malignant central nervous system tumour, accounting for 50.9% of cases in the United States of America between 2016 and 2020 (Ostrom et al. 2023). Survival in patients remains low, with less than seven percent surviving five years post-prognosis and the median survival time in the United States being just eight months (Ostrom et al. 2023). The gold standard of treatment follows the ‘Stupp protocol’ (Stupp et al. 2005), which consists of maximal safe surgical resection followed by radiotherapy plus concomitant and adjuvant chemotherapy using the alkylating chemotherapeutic, temozolomide. Since this landmark change to the treatment of GBM in 2005, moving from sole treatment with radiotherapy to this combined approach and increasing overall survival by 2.5 months, there have been limited changes to in-clinic treatment (Stupp et al. 2005). The most impactful development of GBM therapy has been through tumour treating fields (TTFs) which are low intensity (< 4 V/ cm), intermediate frequency (100–500 kHz), alternating current (AC) electric fields (Stupp et al. 2017; Kirson et al. 2007). When used alongside temozolomide, TTFs have been shown to increase median overall survival by five months, a significant improvement in the context of GBM treatment (Ballo et al. 2023). TTFs were licensed by the United States Food and Drug Administration for recurrent GBM in 2011 and newly diagnosed GBM in 2015 (United States Food and Drug Administration 2011, 2015).

While the biological and biophysical mechanisms of TTFs on cancer cells are extensive and varied, the most fundamental mechanism of action lies in the antimitotic effects of TTFs (Kirson et al. 2007; Rominiyi et al. 2021). During the metaphase of cell division, the alignment of dipoles of molecules, such as microtubules involved in spindle formation, with the uniformly distributed electric fields causes abnormal cell division and cell death by apoptosis. In anaphase, the non-uniform electric field leads to dielectrophoresis of polarisable molecules. This results in the migration of the charged particles to the cleavage furrow, interfering with cytokinesis (Kirson et al. 2004; Giladi et al. 2015). For spherical cells, such as a non-mitotic cell or a cell in metaphase, orientation to the electric field is irrelevant. However, as the cell shape becomes irregular, the angle of the electric field and the divisional axis of the cell affect the effect of TTFs on cell survival. Maximal intracellular field intensity is observed when the axis of the division of a cell in telophase is parallel to the electric field and decreases as the angle tends to 90°. Here, when the cell is orthogonal to the electric field, the maximal intracellular field intensity is at its minimal value (Wenger et al. 2018; Tuszynski et al. 2016).

It has previously been shown that the efficacy of TTFs can be adjusted by controlling the frequency of the AC fields, depending on the type of cells (Branter et al. 2022; Kirson et al. 2007). The glioblastoma cell line, U87, has the highest percentage of cell death when exposed to TTFs at a frequency of 200 kHz, while for patient-derived glioblastoma cells GIN (Glioma INvasive margin) 31 and GCE (Glioma Core Enhanced) 31 cells, a frequency of 200—300 kHz was most effective. The optimal frequency for TTFs treatment is variable, however it is hypothesised to be inversely linked to cell size and the dielectric properties of different cell types (Kirson et al. 2007; Wenger et al. 2018). The duration of electric field application also influences the efficacy of TTFs, with improved outcomes seen in patients who use the device for more than 18 h per day (Ram et al. 2021). This is because cell division is only disrupted when the cells are exposed to the TTFs.

A clear clinical need in GBM treatment focuses on the enhancement of TTFs, with the use of inorganic nanoparticles (NP) being suggested to achieve this (Yoon et al. 2020; Jain et al. 2022, 2024). By capitalising on the major mechanisms of action, which include dipole alignment and dielectrophoresis, it was shown that barium titanate, gold, zinc oxide (ZnO) and silica NP all enhanced the TTFs effect.

To investigate the effects of TTFs in vitro, Novocure™ designed the research system ‘inovitro™’, consisting of eight ceramic dishes housed in a base plate connected to a generator (Novocure 2013; Porat et al. 2017). This device has the advantage of maximising the area of the cells exposed to an electric field by using two pairs of electrodes found perpendicular to each other within the base of each dish. Consequently, a greater number of cells are exposed to the electric field. This is because a higher proportion of cells will randomly align in the correct orientation to one set of electric fields, resulting in maximum treatment efficacy.

Despite the Novocure™ platform being effective, challenges associated with device cost, along with the limited ability to track the effect of the TTFs in real time due to the opaque base of the dish have resulted in efforts to engineer additional platforms that can mimic the effects of AC electric fields shown by the inovitro™ system (Kirson et al. 2004; Smothers et al. 2023; Giladi et al. 2014a, b; Ravin et al. 2020; Jo et al. 2019). By designing more platforms capable of delivering AC electric fields, broader research into the mechanism and enhancement of AC electric fields can also be achieved by making the technology more widely accessible. A significant amount of research has focused on designing alternate electrical stimulation platforms and has centred on intratumoral modulation therapy. This is when electrodes are implanted into the tumour to deliver electric fields directly at the tumour site (Iredale et al. 2020, 2023). To understand electric field distribution, in silico modelling of electric fields has also been used to map the intensity distribution profiles of the fields (Nguyen et al. 2024; Berkelmann et al. 2019; Wenger et al. 2015).

While work has centred on the mechanisms of dipole alignment and dielectrophoresis, there has been little investigation experimentally into how cells with differing morphologies respond to AC electric fields. It is therefore of interest to use a platform that allows for high throughput to better understand the AC electric field mechanisms of action. An AC plate designed for cardiomyocyte work (V. Nguyen, unpublished) has been adapted for this work to monitor changes in cell metabolism based on exposure to either electric fields or currents.

## Methods

### Reagents and materials

Dulbecco’s Modified Eagle Medium (DMEM), foetal bovine serum (FBS), phosphate buffered saline (PBS), antibiotic-antimycotics, and L-glutamine were purchased from Gibco by ThermoFisher Scientific (Waltham, MA, USA). PrestoBlue™ cell viability reagent was purchased from Invitrogen™ by ThermoFisher Scientific (Waltham, MA, USA). Trypan Blue solution (0.4%) and ZnO NP dispersion (< 100 nm, 20 wt. % in H_2_O) were purchased from Sigma Aldrich (St. Louis, MO, USA).

### Cell culture

GIN 31 (Glioma INvasive margin) cells were isolated from the 5-aminolevulinic acid (5ALA) fluorescence positive margin and GCE 31 (Glioma Core Enhanced) cells were isolated from the core central region of a GBM patient at the Queen’s Medical Centre, University of Nottingham (Nottingham, UK) using the method described by Smith et al. (2017) Cells were maintained in DMEM supplemented with 10% (v/v) FBS, 100 U/ mL penicillin, 100 μg/ mL streptomycin, 0.25 μg/ mL Amphotericin B, and 2 mM L-glutamine (Complete DMEM). The cells were kept in an incubator at 37 °C and 5% CO_2_ and were washed using phosphate buffer saline (PBS) and then passaged using trypsin–EDTA when confluent (80%). The cells used were between passages 30–40. D425 (Med Human Medulloblastoma) cells were maintained in DMEM supplemented with 10% FBS. Before passaging, cells were washed using Hank’s Balanced Salt Solution (HBSS) and then trypsinised as above. All cells were tested monthly for mycoplasma where they were grown in an antibiotic-free medium for one week before mycoplasma testing. All cells used were mycoplasma-free.

### AC electrical stimulation platform

**Fig. 1:**
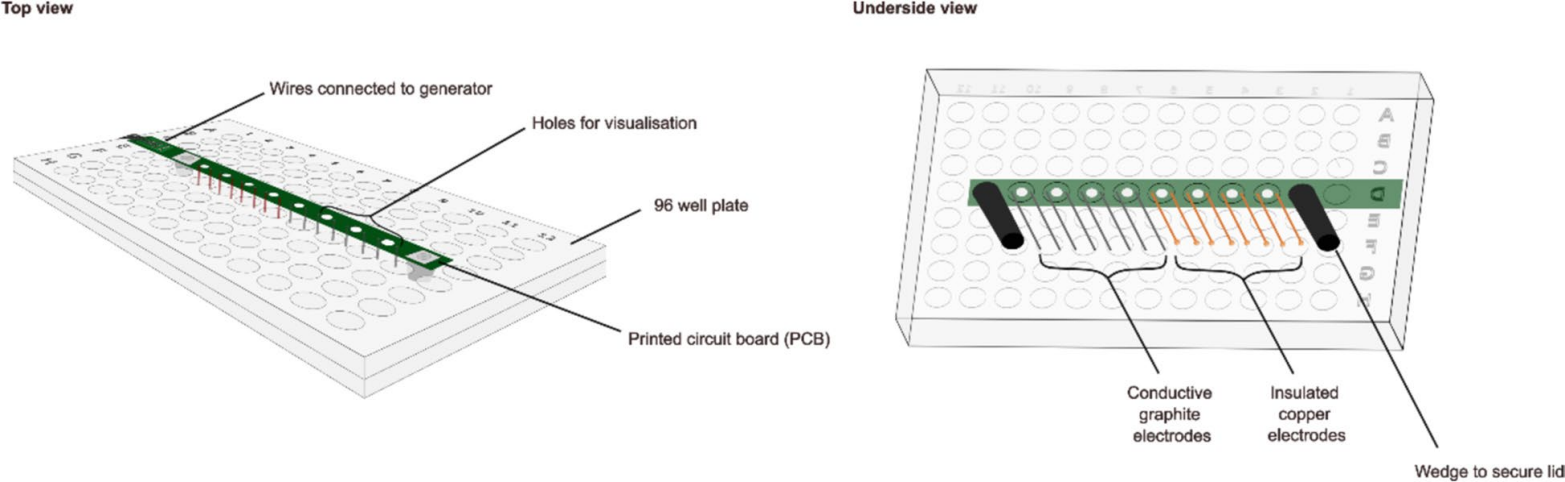
Setup of the modified 96-well plate for AC electrical stimulation. The left-hand diagram shows the setup from the top side of the lid attached to the plate; the right-hand diagram shows the underside of the 96-well plate lid housing four sets each of conductive graphite electrodes and insulated copper electrodes

A Corning™ Costar™ 96-well cell-culture treated plate by ThermoFisher Scientific (Waltham, MA, USA) was modified to include a printed circuit board (PCB) housing four sets of insulated copper wire electrodes to test for electric field effects, and four sets of conductive graphite electrodes to test for electric current effects (See Fig. 1). The electrodes were placed 5 mm apart in each well to ensure that the maximum proportion of cells were exposed to electrical stimulation. In this way, most of the cells were centrally located between the electrodes. Both electrode types were cylindrical, with the main difference between them coming from the material (copper wire and graphite) and the coating of the copper wire tip with a resin to ensure they were fully insulated.

### AC electrical stimulation application

Cells were seeded in a cell culture-treated 96-well plate and incubated for 24 h at 37 °C and 5% CO_2_ to allow for cell attachment. The cell medium was replaced with complete DMEM, and the plate lid was replaced with the AC-modified lid. Cells were incubated at 25 °C for 4, 24 or 48 h with electrical stimulation at 300 kHz and 3 V/ cm in line with previous work (Jain et al. 2022). The input signal was a sinusoidal wave with a frequency of 300 kHz, an amplitude of 3 V/cm, and an impedance of 50 Ω. The signal was applied without any offset or phase shift. After stimulation for the desired length of time, cell media was replaced with 10% PrestoBlue™ cell viability agent from ThermoFisher Scientific (Waltham, MA, USA) and incubated at 25 °C for 3 h before being transferred to an opaque black 96-well plate (Nunc™; ThermoFisher Scientific (Waltham, MA, USA)). The fluorescence was read using a TECAN Infinite 2000 plate reader (Männedorf, ZH, CHE)) at an excitation wavelength of 560 nm and emission of 590 nm. The data was normalised against a negative control of cells without electrical stimulation and a positive control of cells without stimulation treated with 3% TX-100 from Sigma Aldrich (St. Louis, MO, USA)). This is recorded in all figures as the ‘Relative change in metabolism (%)’.

### AC electrical stimulation enhancement

For adherent cell lines, cells were seeded in a cell culture-treated 96-well plate and incubated for 24 h at 37 °C and 5% CO_2_ to allow for cell attachment. Cell media was then replaced with media containing 5 µg/ mL ZnO NP and incubated for 4 h at 37 °C and 5% CO_2_ to allow for cellular uptake (Jain et al. 2022). Media was replaced with complete DMEM and cells were electrically stimulated as described in “*AC Electrical Stimulation Application*” for 48 h. Relative metabolism was determined as described in this previous section.

For semi-adherent cell lines, cells were seeded as above and incubated for 24 h to allow for partial adherence. To prevent the removal of adherent cells, the plate was first spun at 123 × *g* for 5 min. The supernatant was then replaced with media containing 5 µg/ mL ZnO NP and cells were incubated as above. After 4 h, the plate was spun at 123 × *g* for 5 min and the media was replaced with supplemented DMEM. Cells were electrically stimulated as described previously. After 48 h, 10% PrestoBlue™ was added to each well to determine the relative metabolic activity.

### Statistical analysis

All statistical analysis was performed in GraphPad Prism v 10.3.0 software (GraphPad Software, Inc.). All data is expressed as mean ± either standard error of the mean (SEM) or standard deviation (SD). The number of biological repeats is expressed by ‘N’, while the number of technical replicates is expressed by ‘n’. A two-way analysis of variance (ANOVA) with a Tukey multiple comparison post-test was used and p-values of ≤ 0.05 were considered as significant.

## Results & discussion

**Fig. 2:**
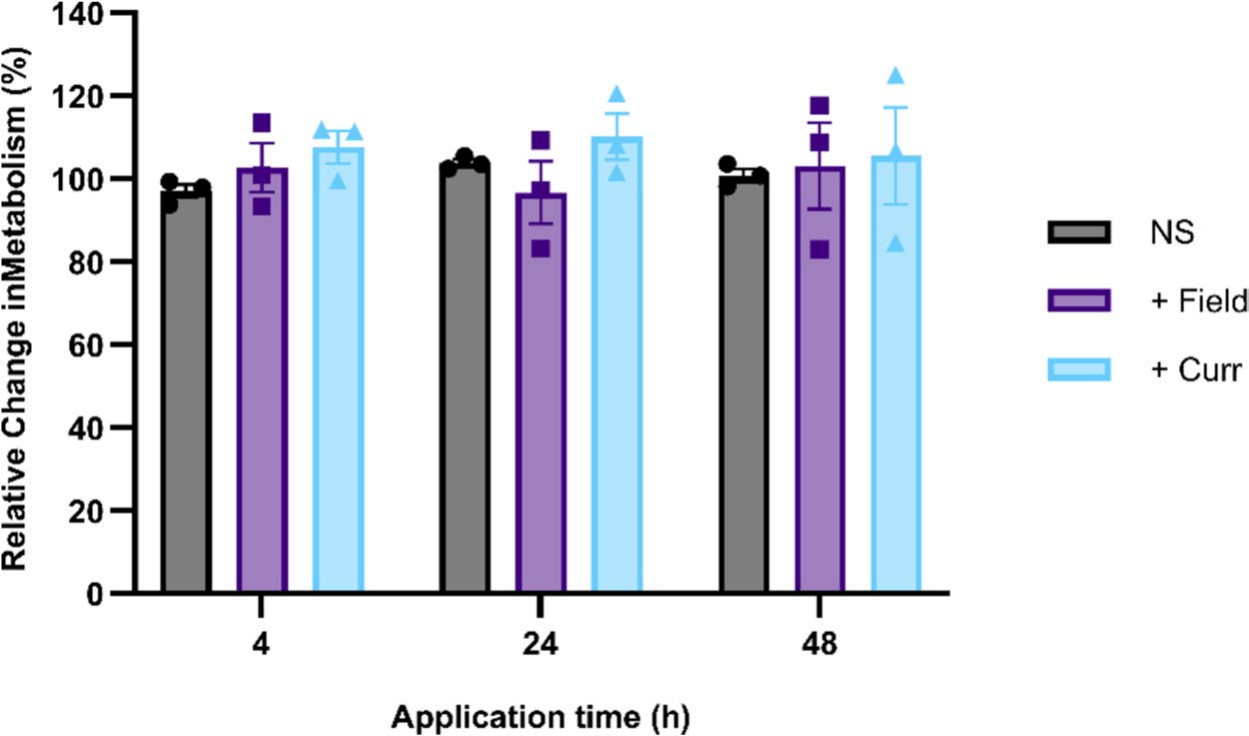
Initial study of 96 well-plate electrode platform. GCE 31 cells were either non-stimulated (NS) or exposed to insulated copper electrodes to observe field effects (+ Field) or conductive graphite electrodes to observe current effects (+ Curr). Metabolic activity of GCE 31 cells was monitored after 4, 24 or 48 h of treatment. *N* = 3, *n* = 1; results represent mean ± SEM

To study the optimum application time using the AC electrical stimulation platform, GCE 31 cells were seeded in a 96-well plate and incubated to allow attachment to the plate surface. Next, AC electric fields or currents were applied at a frequency of 300 kHz and 3 V/ cm, based on the optimum conditions previously identified when using TTFs (Jain et al. 2022; Branter et al. 2022), for either 4, 24 or 48 h (Fig. 2). It was hypothesised that as the application time increased, the relative cell metabolism would decrease as death occurs during mitosis.

Interestingly, while the standard error of each control expanded with increasing electrical stimulation application time, there was no significant difference seen between the negative control and either the cells exposed to the electric field (insulated electrode) or electric current (conductive electrode). While it is well known that TTFs do not affect astrocytes or other non-cancerous cells (Branter et al. 2022), the lack of impact seen using the AC electrical stimulation platform on glioma cells here contradicts the outcomes seen in many other in vitro platforms (Giladi et al. 2014a, b; Voloshin et al. 2020).

**Fig. 3:**
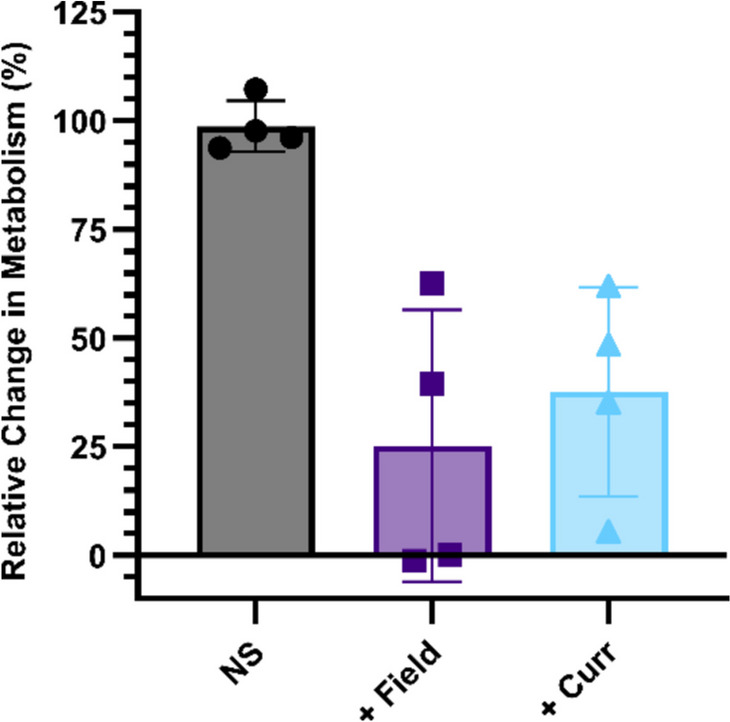
Relative metabolism of GCE 31 cells without stimulation (NS)) or 48 h exposure to AC electric fields (+ Field), or current (+ Curr) at increased temperature (37°C). Results show mean ± SD; *N* = 1, *n* = 4

To increase the responsiveness of the cancer cells to the electrical stimulation, we therefore decided to incubate the cells at a higher temperature. This is because cell growth rate increases as the temperature approaches physiological conditions, so we hypothesised that a more pronounced electric field effect would be observed when the cells are incubated at 37 °C (Giladi et al. 2015; Moore et al. 1997). Increasing the temperature could result in more cells in both the mitotic stage of the cell cycle and in the correct orientation for the electric fields to induce an effect (Moore et al. 1997). However, at this higher temperature, localised heat generated by the electric fields resulted in the evaporation of water and subsequent increased concentration of salts (Porat et al. 2017). For the electric fields, the change in salts may have led to a decreased relative permittivity and increased electric field strength. For the current effects, the increased salt concentration is likely to have resulted in reduced electrical resistivity and increased current (Fig. 3) (Lubbe et al. 2023; Widodo et al. 2018). In both instances, we hypothesise that the decrease in cell metabolism is due to the changed makeup of the cell culture medium, not due to intracellular effects of electric fields or currents. Therefore, to ensure the effects observed were due to intracellular effects, all future experiments were conducted in an incubator set at the lower temperature of 25 °C.

As the electrical stimulation alone did not alter cellular metabolic activity in this work, we sought to amplify their effect. Previous studies conducted in our group found that three different types of inorganic NP, (ZnO, silica, and gold) when coupled with TTFs, reduced the metabolic activity of GBM cells (Jain et al. 2022). Therefore, ZnO NP were chosen as the electric field amplifier for this study. As with our previous study, here cells were incubated with the NP at a non-toxic concentration of 5 µg/ mL for 4 h to allow for cellular uptake, after which the cell medium was replaced to remove any NP not within the cells. AC electric fields or currents were then applied for 48 h to, again, maximise any potential effects of the fields on the cells.

**Fig. 4:**
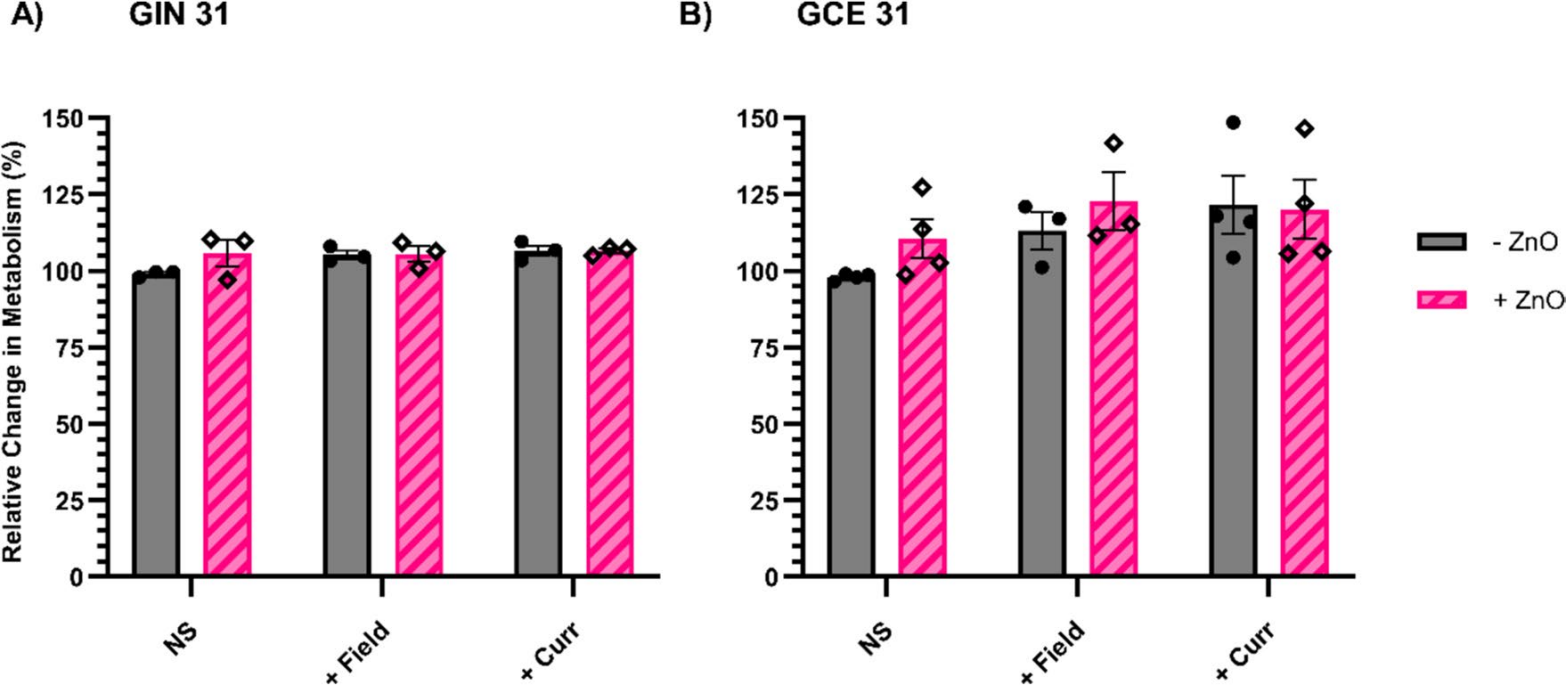
Changes to metabolic activity of GIN31 cells (**A**) and GCE31 cells (**B**) without electrical stimulation (NS) or following 48 h exposure to AC electric fields (+ Field) or current (+ Curr), in the presence or absence of ZnO. Cells were treated with ZnO NP for 4 h before electrical stimulation; the NP were then removed and replaced with fresh cell culture medium. *N* = 3/ 4, *n* = 2/ 4. Results show mean ± SEM

Despite the significant decrease in metabolic activity previously seen when using the inovitro™, there was no significant change when GIN 31 cells (taken from the invasive margin of the GBM tumour) were exposed to AC electric fields or currents with or without ZnO NP (Fig. 4A), indicating the challenges associated with the setup used here. Increasing metabolic activity trends were observed for GCE 31 cells (taken from the core of the GBM tumour) for all treatments, however this was not shown to be a significant increase (Fig. 4B).

**Fig. 5:**
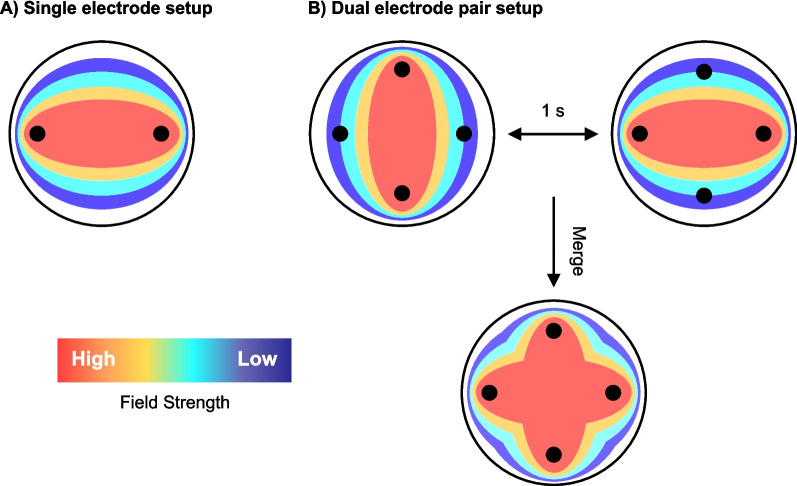
Schematic drawing showing electric field distribution of two different electrode setups, where red shows the highest electric field strength and dark blue shows the lowest electric field strength. **A** Single electrode setup where electric field orientation remains constant between the single pair. **B** Dual electrode setup where electric field orientation changes orientation by 90° every 1 s. Merged electric field distribution combining both orientations shown

Given the lack of effects observed when adherent cells were treated with electric fields, it was important to consider the orientation of the electric fields in this platform, as field directionality greatly impacts the efficacy. Mitotic cells are only impacted by the presence of an electric field when they are in alignment with the field. Therefore, by only using one pair of electrodes to generate the field effect, we hypothesised that the number of cells affected would be limited to the number of cells in the correct orientation (Fig. 5A) (Pfeifer et al. 2021; Berkelmann et al. 2019). To overcome this barrier, other work has used at least two pairs of electrodes, perpendicular to one another, with a frequency phase shift as described by (Iredale et al. 2023) to better replicate the TTFs seen using the inovitro™ setup (Fig. 5B).

**Fig. 6:**
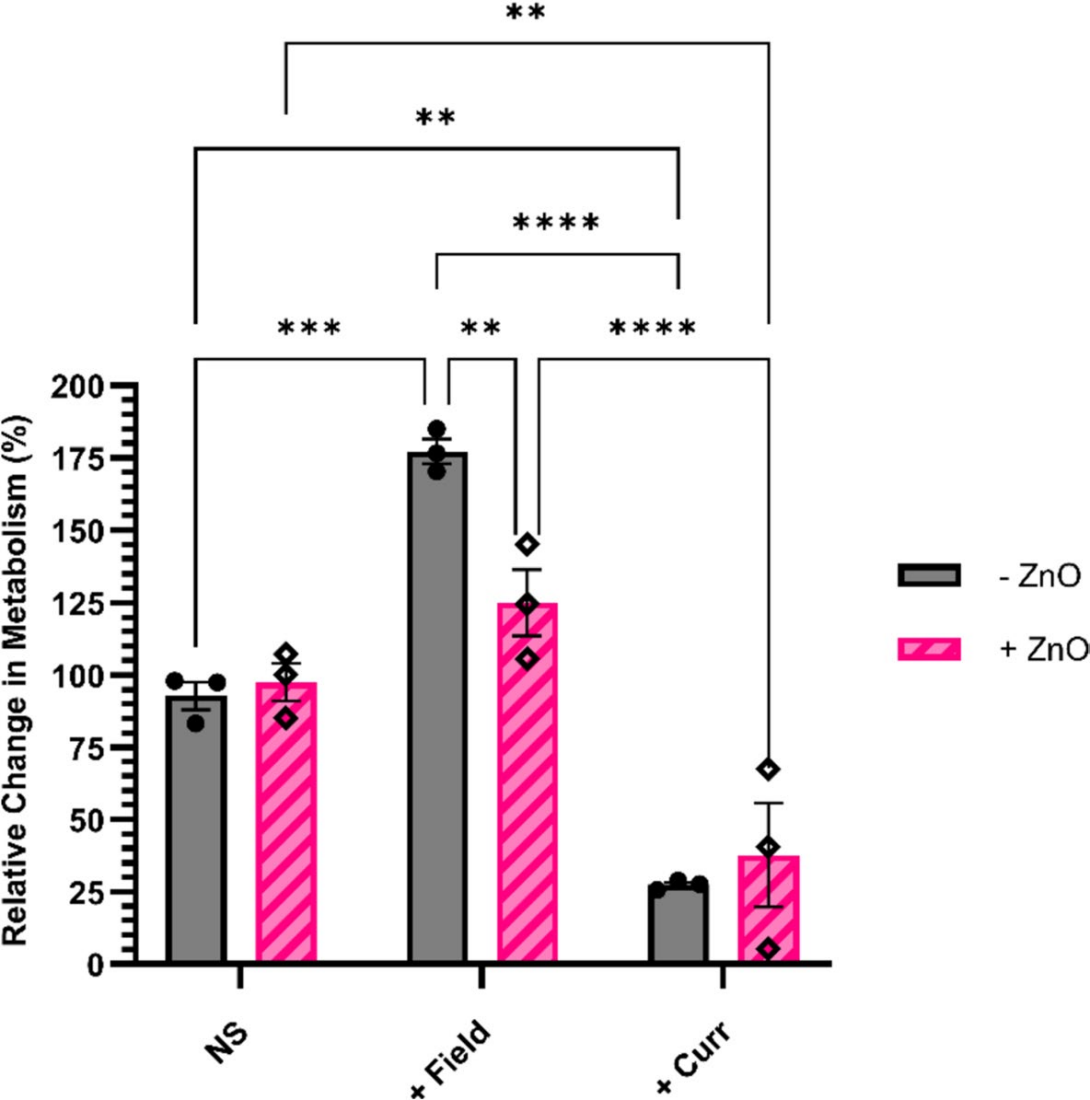
Changes to metabolic activity following 48 h exposure to AC electric fields (+ Field) or current (+ Curr) at increased temperature (37°C), in the presence or absence of ZnO, on D425 cells in comparison to non-stimulated (NS) cells. Cells were treated with 5 µg/ mL ZnO NP for 4 h before electrical stimulation; NP were then removed and replaced with fresh cell culture medium. *N* = 3, *n* = 2. Results show mean ± SEM; **P = 0.0021, ***P = 0.0002, ****P < 0.0001

To confirm the hypothesis that the unilateral electrode orientation limits the proportion of cells exposed to the electric fields, a semi-adherent cell line was chosen, as there would be a higher proportion of cells randomly in the correct alignment with the fields. Therefore, the cell line D425 (paediatric medulloblastoma) was used. Using a modified protocol to the GIN and GCE 31 adherent cells, the effects of electric fields or current with or without ZnO NP were investigated (Fig. 6). Here the relative metabolic activity of cells exposed to AC electric fields was markedly increased to 177% of the unstimulated (negative) control. While there was an increase in the relative metabolism of cells treated with both electric fields and ZnO NP, this was not found to be significantly different from cells solely treated with ZnO NP (*P* = 0.1417). Conversely, for the cells treated with current in either the presence or absence of ZnO NP, there was a significant decrease in the relative metabolism (38% and 27% of the controls without stimulation or NP, respectively).

These results greatly differ from those seen by both the GIN 31 and GCE 31 adherent cells. While the decrease in metabolic activity upon treatment with radiofrequency AC electric currents is well-documented (Hernández-Bule et al. 2019), the increase of metabolic activity upon treatment with AC electric fields is not. Liu et al*.* have previously shown that morphology, spherical or elongated, has a pronounced effect on the distribution of the electric fields across a single cell (Li et al. 2020). The findings in this work corroborate their conclusions that the mechanisms of action of electric fields on cancer cells are not well understood. Based on the difference in metabolic activity of the adherent and semi-adherent cell lines, we hypothesise that the cell size and shape has an impact on the responsiveness of cells to AC electric fields.

## Conclusions

This study highlights the importance of further investigating the mechanisms of action of AC electric fields on different cancer cells. A high-throughput modified 96-well plate consisting of one pair of electrodes per well was used to test a simple in vitro setup. After finding no significant difference in metabolic activity of a patient-derived GBM cell type after AC electric field or current application for 4, 24 or 48 h, we aimed to test if combining this electrical stimulation with ZnO NP would improve the observed efficacy of the platform. Contrary to previous literature, it was found that there was no significant change in the metabolic activity of GIN 31 and GCE 31 cells when treated with both 5 µg/ mL ZnO and AC electric currents at a frequency of 300 kHz and 3 V/ cm. Contrasting this, the semi-adherent D425s showed a significant increase in metabolic activity when exposed to electric fields alone, and significant decreases in metabolic activity when exposed to current treated with or without ZnO NP.

Much focus of the mechanism of action of TTFs lies around the interference of spindle formation and movement of internal molecules by dipole alignment and dielectrophoresis. While electric field directionality is important in inducing cell cycle arrest, in this paper we highlight the need to understand how different cancer cells respond to AC electric fields. Further research into the effect of cell morphology on electric field responsiveness is therefore needed to enable the alternating electric fields anticancer therapy to be enhanced. This would allow for much-needed progression of this therapy to improve patient outcomes.

## Data Availability

All data associated with this manuscript can be found at https://rdmc.nottingham.ac.uk/. DOI to be included on publication.

## Abbreviations

AC: Alternating current
GBM: Glioblastoma
GCE: Glioma Core Enhanced (cell line)
GIN: Glioma INvasive margin (cell line)
NP: Nanoparticle
TTFs: Tumour treating fields
ZnO: Zinc oxide
